# Parasite dynamics in untreated horses through one calendar year

**DOI:** 10.1186/s13071-022-05168-z

**Published:** 2022-02-08

**Authors:** Ashley E. Steuer, Haley P. Anderson, Taylor Shepherd, Morgan Clark, Jessica A. Scare, Holli S. Gravatte, Martin K. Nielsen

**Affiliations:** 1 School of Veterinary Medicine, Texas Tech University, Amarillo, TX USA; 2 College of Veterinary Medicine, Auburn University, Auburn, AL USA; 3 College of Veterinary Medicine, Lincoln Memorial University, Harrogate, TN USA; 4 Department of Animal Science, Eastern Kentucky University, Richmond, KY USA; 5 Department of Veterinary Science, Maxwell H. Gluck Equine Research Center, University of Kentucky, Lexington, KY USA

**Keywords:** Horses, Strongyle, *Strongylus vulgaris*, Cyathostomin, Seasonality, Serology

## Abstract

**Background:**

Horses are host to a plethora of parasites. Knowledge of the seasonality of parasite egg shedding and transmission is important for constructing parasite control programs. However, studies describing these patterns are sparse, and have largely been conducted only in the United Kingdom. This study evaluated strongylid egg shedding patterns and transmission dynamics of *Strongylus vulgaris* in naturally infected and untreated mares and foals through one calendar year in Kentucky, USA. The study also investigated the existence of a peri-parturient rise (PPR) in strongylid egg counts in foaling mares and collected information about *Strongyloides westeri* and *Parascaris* spp. in the foals.

**Methods:**

This study was conducted from January to December 2018. A herd of 18 mares, one stallion, and 14 foals born in 2018 were followed throughout the year. Sera and feces were collected biweekly from all horses, and worm burdens enumerated in 13 foals at necropsy. An *S. vulgaris* ELISA antibody test was run on all serum samples. Fecal egg counts were determined for all horses, and coproculture and qPCR assay were employed to test for the presence of *S. vulgaris* in the mature horses. Data were analyzed using the proc glimmix procedure in the SAS 9.4 software program.

**Results:**

We found a general lack of seasonality in strongylid egg shedding throughout the year among the mature horses, and no PPR was demonstrated. Shedding of *S. vulgaris* eggs displayed a higher abundance during the spring, but findings were variable and not statistically significant. Anti-*S. vulgaris* antibody concentrations did not display significant fluctuations in the mature horses, but evidence of passive transfer of antibodies to the foals was demonstrated, and foals assumed their own production of antibodies starting at approximately 20 weeks of age. Overall, colts shed higher numbers of strongylid, ascarid, and *S. westeri* eggs than fillies.

**Conclusions:**

This study demonstrated a lack of seasonality in strongylid egg shedding for the study population, which is in stark contrast to previous studies conducted elsewhere. This strongly suggests that more studies should be done investigating these patterns under different climatic conditions.

**Graphical Abstract:**

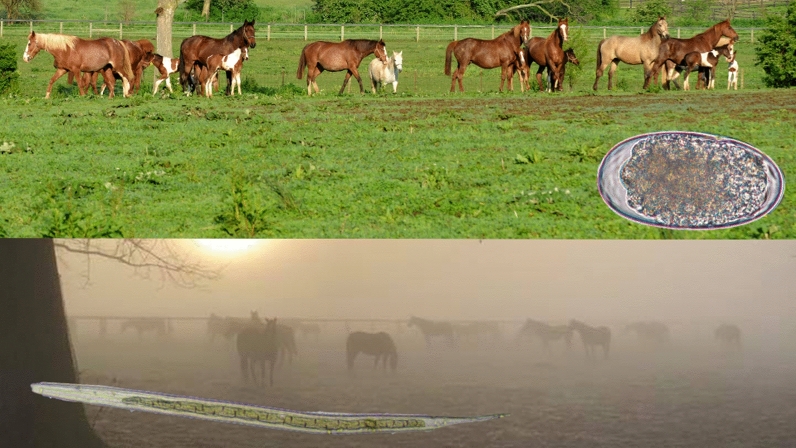

## Background

Equine strongylid parasites are ubiquitous around the world. Widespread anthelmintic resistance is reported in these parasites [1], and more information about their seasonality and epidemiology is needed to assist in determining appropriate approaches for parasite control. The Strongylidae family in equids comprises two subfamilies: Cyathostominae (cyathostomins, small strongyles) and Strongylinae (strongylins, large strongyles). Cyathostomins are commonly reported to show anthelmintic resistance to all currently available drug classes [1, 2], whereas the group of Strongylinae, which includes the bloodworm *Strongylus vulgaris*, has not been reported resistant to anthelmintic products.

*Strongylus vulgaris* is widely regarded as the most pathogenic intestinal helminth parasite in horses [3, 4]. With the advent of modern anthelmintics in the 1960s, an interval dose approach was introduced with a primary aim to control this parasite in equine herds [5]. Frequent anthelmintic treatments administered at regular intervals year-round significantly reduced the prevalence of *S. vulgaris*, and cyathostomins subsequently became the focus of parasite control [6, 7]. However, these decades of intense anthelmintic treatment regimens also led to widespread drug resistance among cyathostomins [1, 2]. Because of this development, current recommendations aim to adopt surveillance-based parasite control programs and reduce anthelmintic treatment intensities to delay further resistance development as much as possible [8–10].

While the development and survival of free-living stages of the life-cycle is well described for all strongylid parasites and has been demonstrated to be dependent on weather, season, and climate [11, 12], the parasitic phases differ substantially between cyathostomins and strongylins. Larvae of *S. vulgaris* migrate in the mesenteric arteries for about 4 months before they return to the intestinal tract and reach sexual maturity at about 6 months of age [13]. In comparison, some cyathostomin parasite species can complete their life-cycle in 6–8 weeks [14], but encysted early third stage larvae (EL3) can undergo arrested development for at least 2 years [15–17]. Thus, epidemiological patterns likely differ substantially between cyathostomins and *S. vulgaris*.

Despite the recognized importance of equine strongylid parasites and the emphasis on surveillance-based approaches to controlling them, a limited number of studies have been published on seasonal patterns of strongylid transmission dynamics in equines, and the majority of these have been conducted in the United Kingdom (UK). In 1954, Poynter [18] was the first to describe seasonality in equine strongylid egg shedding, and reported egg counts to be lowest in the winter and highest during July, August, and September. This was followed up by Duncan in 1974 [11], who also described a seasonal pattern in strongylid egg shedding, with counts increasing during the spring and subsequently plateauing during summer. In the past decade two British studies have been published, with one finding similar seasonal egg shedding patterns [19], while the other found no evidence of such seasonality among adult horses [20]. Recently, a Swedish 2.5-year study of free-ranging ponies found strongylid fecal egg counts (FECs) to peak in August/September [21], and a New Zealand study reported broodmares to exhibit lower strongylid FECs during winter months [22], both confirming Poynter’s observations from almost 70 years ago. One study performed in ponies in Louisiana, USA, reported significantly lower mean strongylid FECs during winter months, but these were data from ponies that were euthanized at different time points, and repeated measures were not done across seasons [23]. Taken together, strongylid egg shedding patterns have not been investigated widely, and very few studies have been reported from outside of the UK.

Strongylid egg shedding patterns have been documented to be influenced by a variety of factors including age, sex, and pasture management. Young horses (1–4 years) have shown elevated strongyle egg shedding patterns [19, 20, 24], while middle aged horses (5–15 years) often display the lowest strongyle egg output [24], and older horses (20–33 years) have been shown to have higher strongyle egg counts [24, 25]. A few studies have found different strongylid egg count levels between male and female horses, but results are conflicting. One study reported higher strongylid FECs in 2–4-year-old domesticated males [24], while a study of feral horses found that males displayed lower strongylid FECs in some locations [26]. A peri-parturient rise (PPR) in strongylid egg shedding has been widely documented in sheep [27, 28]; however, Duncan investigated this phenomenon among nine foaling mares and did not observe any fluctuations [11]. There are no other published studies evaluating the possible existence of a PPR in horses, so more research is warranted before any clear conclusions can be drawn.

Duncan also documented a seasonal pattern in *S. vulgaris* egg shedding density, with elevated coproculture larval counts during the spring and summer compared to the remainder of the year [11], but the seasonality of this parasite has not been widely studied since then. One study evaluated the seasonality and distribution of *S. vulgaris* in foals and documented passive transfer of maternal anti-*S. vulgaris* antibodies [29]. Although it can be assumed that the concentration of these maternal antibodies is a function of the serum antibody concentration of the accompanying dam, this relationship has not been investigated at this stage.

The overall aim of this study was to characterize the seasonality of strongylid type egg shedding over the course of one calendar year in a herd maintained without anthelmintic treatment in central Kentucky, USA. Additional aims were to (1) investigate the possible existence of PPR in foaling mares, (2) describe the seasonality of *S. vulgaris* egg shedding and serum antibody responses in mares and foals, (3) characterize the relationship between anti-*S. vulgaris* antibody concentrations in foals and their dams over the course of the year, (4) determine *Strongyloides westeri* and *Parascaris* spp. egg shedding in the foals, and (5) document adult and larval strongylid and ascarid worm burdens in necropsied foals from the herd.

## Methods

### Study population

This study was conducted from January to December of 2018 in central Kentucky, USA, a warm temperate, fully humid, hot summer climate (Cfa), based on the Köppen-Geiger climate classification system. Horses from the University of Kentucky’s anthelmintic naïve parasitology herd were evaluated under the University of Kentucky’s Institutional Animal Care and Use Committee protocol 2012–1046. This closed herd had not received anthelmintics and has been located on the same grazing pasture since 1979 [30]. Horses were provided free access to pasture, hay, and mineral blocks, and were fed a ration balanced grain supplement over the course of the study. Two age groups were defined within this herd: a mature horse population with ages of 7–18 years (*n* = 19, 18 mares and one stallion) and foals born into the herd in 2018 and followed until necropsy at 4–8 months of age. All mares were bred in the field via live cover in 2017. Foals (*n* = 14; 9 fillies, 5 colts) were born between April and July 2018, and 13 of these were euthanized either at 4–6 months of age (*n* = 9) or at 6–8 months of age (*n* = 4).

### Fecal and serum sample collection

Samples were collected biweekly from all mature horses and weekly/biweekly from the foals, depending on the behavior of the individual animals. Fecal samples were collected from fresh droppings or manually from the rectum. Serum samples were collected by venipuncture of the jugular vein(s) of each animal. Fecal samples were immediately packaged, labeled, and stored at 4 °C for further laboratory processing, and serum samples were stored at − 80 °C until processing.

### Fecal egg counts

FECs were determined in triplicate using the Mini-FLOTAC method as described previously [31], using a saturated glucose-NaCl flotation medium (specific gravity of 1.25) and with a multiplication factor of five eggs per gram (EPG). Specimens were evaluated for strongylid, *Parascaris* spp., and *S. westeri* type eggs. However, due to an abundance of *S. westeri* in the foal samples, *S. westeri* egg counting was discontinued after a threshold of 5000 EPG was reached.

### Coproculture and larval strongylid differentiation

Individual coproculture was carried out for all mature horses upon each collection, using 10 g of fecal matter and an equal amount of vermiculite to promote water retention and aeration, as described previously [32]. Samples were incubated at room temperature for 14 days and then placed in a Baermann apparatus for an additional 2 days. The entire sediment was harvested, and larvae were stored at 4 °C until identification. For identification, larvae were transferred to nematode counting slides and subsequently heat-inactivated by placing the nematode counting slide on a warming plate at 60 °C for 5 min, or until small bubbles formed. Samples were then examined microscopically at ×10 magnification, and third-stage larvae were counted up to 500. Larvae were identified to genus and/or species using a published key [33].

### *Strongylus vulgaris* qPCR

Eggs were isolated from fecal samples via egg isolation methods described previously [34]. Briefly, 10 g of feces was mixed with 50 ml of tap water. The mixture was strained through a two-ply cheesecloth (18 × 36 in., grade 10 mesh, Fisher Scientific, Waltham, MA, USA) and centrifuged for 10 min at 300×*g*. The pellet was then suspended in 50 ml of glucose-NaCl flotation solution (SG = 1.25) and centrifuged again at 300×*g* for 10 min. The supernatant containing the eggs was then subjected to straining through a series of filters arranged by filter size (largest to smallest: 200 μm, 100 μm, and 27 μm) (pluriSelect Life Science, Leipzig, Germany). Isolated eggs were then retrieved from the 27 μm filter and stored in 100% EtOH at −20 °C until DNA isolation. Ethanol was removed from egg samples prior to DNA extraction via evaporation. The Quick DNA Fecal/Soil Microbe kit (Zymo Research, Irvine, CA, USA) was used to isolate DNA from egg samples according to the protocol provided by the manufacturer. Real-time polymerase chain reaction (qPCR) assay was used to identify *S. vulgaris* DNA in isolated eggs as described previously [35]. A mixture of Brilliant II QPCR Master Mix (Agilent Technologies, Santa Clara, CA, USA) and 4 μl of extracted DNA was used, and the temperature and cycles were set as described previously [35]. Primers and probes were obtained from Integrated DNA Technologies (Skokie, IL, USA), and concentrations were set as described previously [35]. Positive controls for the assay were obtained from an adult morphologically identified *S. vulgaris* specimen. For statistical analysis, all negative PCR reactions were recorded as a cycle threshold (Ct) of 100.

### *Strongylus vulgaris* ELISA

Serum samples were measured for concentration of antibodies specific to the SvSXP antigen produced by migrating *S. vulgaris* larvae using an enzyme-linked immunosorbent assay (ELISA) as described previously [36]. All samples were diluted 1:50 with phosphate-buffered saline solution with Tween (PBST) (1:10 dilution), and a secondary antibody of horseradish peroxidase conjugated to goat anti-horse IgG(T) (Bethyl Laboratories, Inc., Montgomery, TX, USA) at a dilution of 1:40,000 was added to each well. Results were normalized as a percentage of the positive control to reduce inter-assay variability [36]. The positive control sample was obtained from a horse known to be infected with *S. vulgaris*.

### Necropsy procedures

The foals were divided into two groups based on a previously reported biphasic appearance of ascarid worm burdens and egg shedding [37, 38], with nine foals (7 fillies, 2 colts) euthanized at the presumed peak ascarid burden age (4–6 months) and four foals (1 filly, 3 colts) euthanized at 6–8 months of age. Necropsy procedures followed previously published principles [39], and the following parasite species and stages were collected and enumerated. Attempts were not made to obtain specimens of *S. westeri* from the small intestines, as egg shedding patterns had indicated that populations of *S. westeri* were eliminated prior to euthanasia.

#### Cyathostomins

Encysted larval stages were enumerated for each of the large intestinal sections—cecum, ventral colon, and dorsal colon—using a mucosal digestion technique as described previously [40], and larvae identified to either early third stage (EL3) or developing stages [late third-stage (LL3) and mucosal fourth-stage larvae (L4)]. A multiplication factor of 500 per organ was used to estimate the total mucosal burden of each organ.

Luminal cyathostomin stages were enumerated for each large intestinal organ by examining a subsample representing a 1% aliquot of the total content volume and subsequently multiplying the count by 100 to estimate the total luminal cyathostomin burden for each foal.

#### *Strongylus* spp.

The cranial mesenteric (CMA) and celiac arteries were dissected, and migrating stages of *S. vulgaris* were collected and morphologically identified to stage (L4 and L5) and sex (L5 only). Similarly, the ventral abdominal walls and peri-renal fat tissues were inspected and dissected for the presence of migrating *S. edentatus* larvae, which were identified to stage (L4 and L5) and sex (L5 only) as well. Intestinal *Strongylus* spp. specimens were collected from the cecum, ventral colon, and dorsal by visual inspection of mucosal walls for attached parasites and macroscopical examination of the entire intestinal content.

#### Other parasites

*Parascaris* spp. specimens were collected by visual inspection of the entire small intestinal tract and by examining the intestinal contents from the large intestines. Specimens were identified to L4 or L5 (male and female), where appropriate. Ascarids in this documented herd have been karyotyped previously and identified as *P. univalens* [41], but karyotyping was not carried out in this study, so findings are reported as *Parascaris* spp. herein. *Anoplocephala perfoliata* were collected by inspection of the cecal mucosal walls and examination of the intestinal contents from the large intestines.

### Statistical analyses

McNemar’s test was run comparing the qualitative results obtained with the coproculture and qPCR using online software (www.graphpad.com). All statistical models were run with SAS University edition software (Cary, NC, USA), and were analyzed with generalized mixed linear models using the glimmix procedure, with a Gaussian distribution assumed. Akaike information criterion and Akaike information criterion corrected were used to assess model fit. JMP Pro 14 software (Cary, NC, USA) was used to create figures and estimate confidence intervals and correlations. Associations of all measured parameters for each analysis was evaluated using traditional backward and forward elimination of variables. All variables with *P*-values < 0.25 were kept in the model. When variables were significant, a least-square means for Tukey’s pairwise comparison, odds ratio, and estimate were all performed, and interpretation of results carried out at a significance level *α* = 0.05.

#### Mature horses

Mature horses were categorized based on age, sex, egg shedding category, and parturition status. At each collection time point, horses were assigned to the following egg shedding categories: low shedders (0–99 EPG), moderate shedders (100–499 EPG), and high shedders (> 500 EPG). Furthermore, the horses were assigned to one of two age categories: 7–11 years (n = 7) and 12–18 years (*n* = 12). Each sample collection time point was assigned a corresponding collection number (1–24) and season: winter (December–February), spring (March–May), summer (June–August), and autumn (September–November). At each time point, mares were assigned a parturition status of either 0 (no foal) or 1 (foal). Models were constructed with FEC, ELISA, PCR, and coproculture results (% *S. vulgaris* and total *S. vulgaris* larvae counted) as outcome variables and with age, season, collection date, and parturition as input variables. “Horse_ID” was kept as a random effect, and repeated measures used with collection date, where appropriate.

#### Foals

In foals, *S. vulgaris* ELISA, FEC (strongylid, ascarid, *S. westeri*), and worm counts (*S. vulgaris*, *S. edentatus*, cyathostomins, *Parascaris* spp.) were analyzed as output variables. In addition to the ELISA values determined at each time point, a foal/mare ELISA ratio variable was created for each foal at each time point as the ratio between the ELISA value in the foal at the time of determination divided by the ELISA value of the corresponding dam at the time of parturition. Models were constructed analyzing these output variables with age, sex, or time point evaluated as input variables. “Horse_ID” was kept as a random effect, and repeated measures used with collection date, where appropriate.

## Results

### Mature horse fecal egg counts

**Fig. 1:**
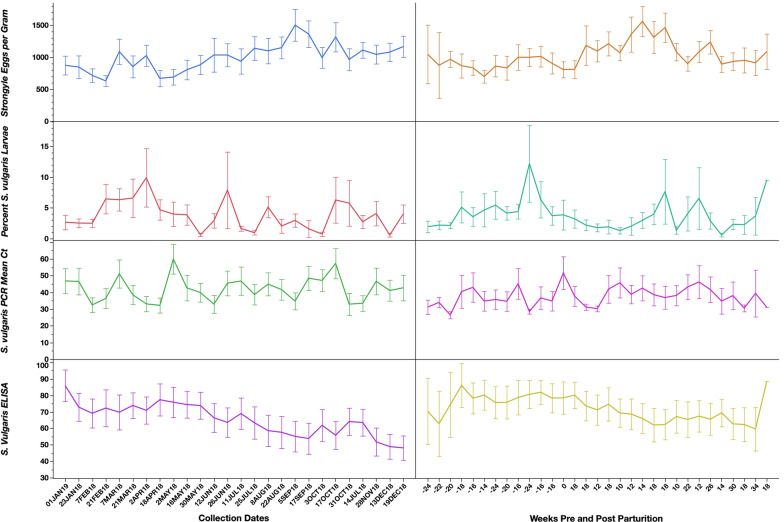
Strongylid fecal egg counts, coproculture results (% *S. vulgaris* larvae), *S. vulgaris* qPCR results, and *S. vulgaris* serum ELISA results for the mature horses throughout the study. Graphs on the left present data by sampling date, whereas the graphs on the right present the data by week from parturition for the mares. *Y*-error bars represent 95% confidence intervals

Mean mature horse strongylid egg counts are presented in Fig. 1. There were no statistical associations between mean strongylid FECs and age groups, seasons, collection dates, or parturition status among the mature horses.

### Foal fecal egg counts

#### Strongylids

**Fig. 2:**
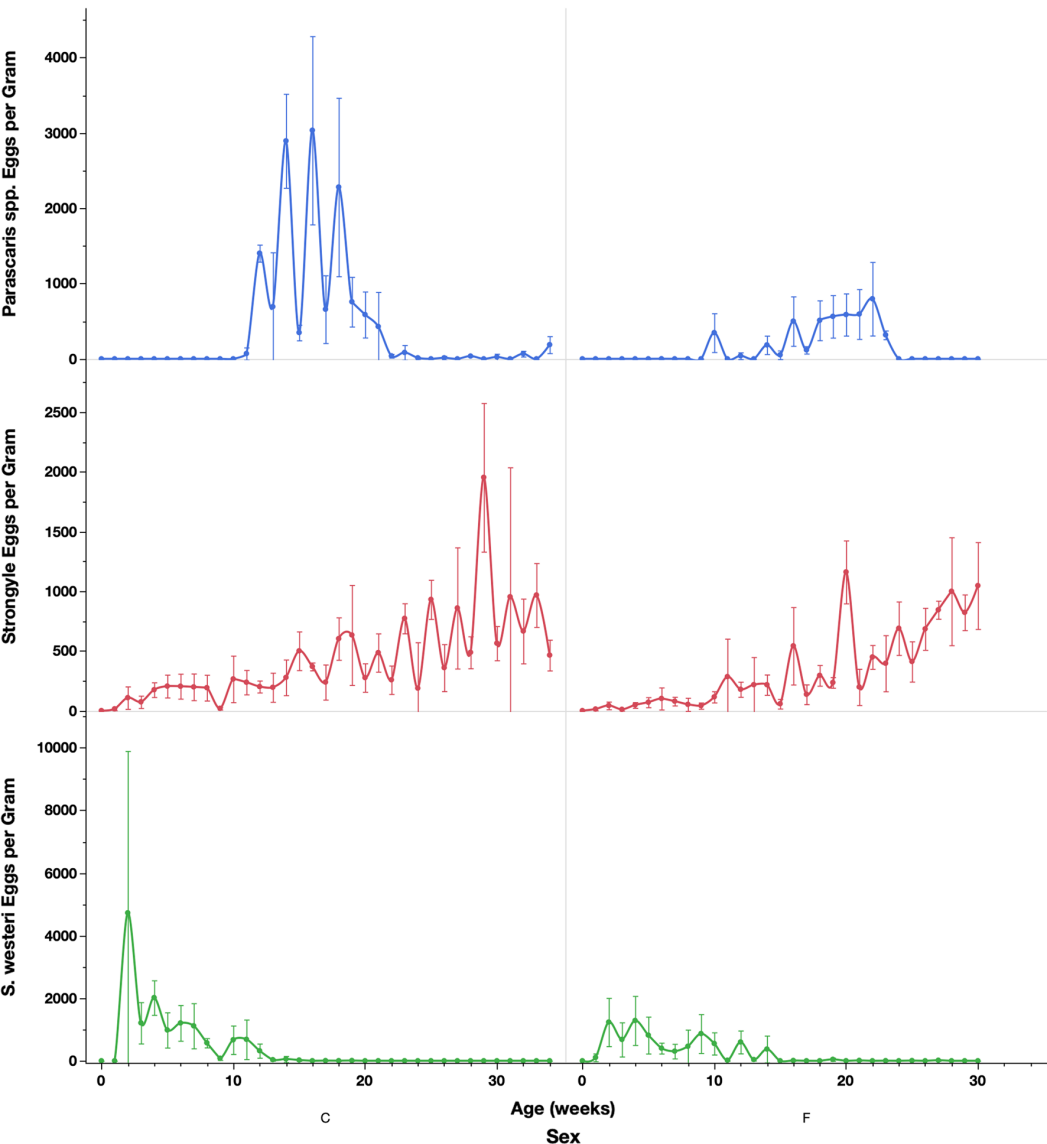
*Parascaris* spp., strongylid, and *S. westeri* fecal egg counts in the foals across the weeks of the study. Graphs on the left present data for the colts, while graphs on the right present data for the fillies. *Y*-error bars represent 95% confidence intervals

For strongylid egg shedding, eggs were first detected in the feces at 1 week of age (Fig. 2). There was a small peak around 6 weeks of age, followed by an increase over time in both colts and fillies (*P* = 0.0163). Colts had a higher peak at6 weeks and a higher count overall (*P* = 0.0152, Fig. 2). Overall, older foals shed significantly higher numbers of eggs, and higher strongylid shedding occurred in the winter than during other seasons (*P* = 0.0208). There was also a significant association with the interaction terms age × season and age × sex, where older foals and colts shed more in the winter season versus other seasons (*P* = 0.0437).

### *Parascaris* spp.

Fecal *Parascaris* spp. shedding started at 6 weeks of age and continued through 37 weeks (Fig. 2). Colts had higher egg shedding than fillies, and peak shedding occurred at 16 weeks for the colts compared to 22 weeks for the fillies; however, this was not statistically significant. When considering age and season, the FECs were significantly higher during the autumn compared to winter, after accounting for the influence of age (*P* = 0.0351). Fillies shed eggs earlier and for a longer duration (11–24 weeks) compared to colts, which shed higher numbers but for a shorter period (10–21 weeks).

#### *Strongyloides westeri*

**Fig. 3:**
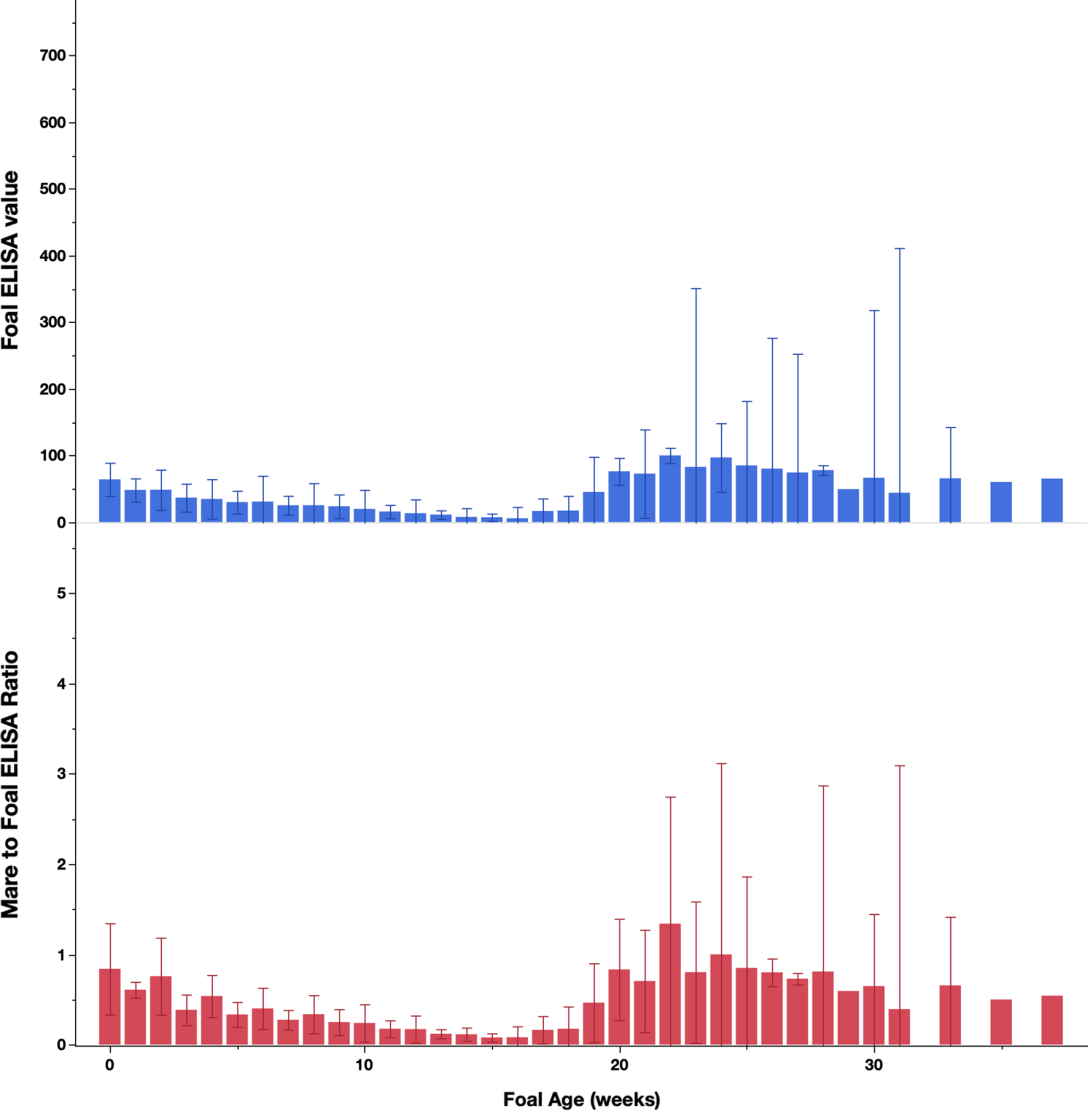
Serum *S. vulgaris* ELISA results from the foals across the study. Top graph presents the results from the foals, whereas the bottom graph presents the ratios between ELISA results from foals at each time point and their dams measured at the week of parturition. *Y*-error bars represent 95% confidence intervals

Age, season, age × season, and age × sex were all significantly associated with *S. westeri* egg shedding (Fig. 2). Peak *S. westeri* shedding occurred at 2 weeks of age and subsequently declined throughout the study; however, the majority of shedding ended by 14 weeks of age. Colts shed higher numbers of eggs during peak shedding, but fillies shed for a longer duration (Fig. 3); however, this was not statistically significant.

### Coproculture

Coproculture results are presented in Fig. 1 and Table 1. Table 2 presents the results of the McNemar test for comparisons between the coproculture and PCR results. Overall*,* the adult horses tested positive for *S. vulgaris* in 68.3% of the coprocultures analyzed. There were no statistical associations between *S. vulgaris* larval counts or percentage and time points or seasons; however, horses in the 12–18 year-old group shed higher total *S. vulgaris* compared to the 7–11 year-old group (*P* < 0.0001).

**Table 1:** The number of adult horses testing positive and negative for *Strongylus vulgaris* by larval culture and PCR across the 26 time points in the study

Sample date	Larval culture		PCR	
	Positive	Negative	Positive	Negative
Jan 1	13	6	15	4
Jan 23	14	4	14	4
Feb 7	15	4	17	2
Feb 21	15	4	18	1
Mar 7	15	4	17	2
Mar 21	14	4	13	5
Apr 2	16	3	18	1
Apr 18	17	2	18	0
May 2	14	5	14	3
May 16	13	5	11	7
May 30	9	10	16	3
Jun 12	15	4	15	4
Jun 26	13	5	15	3
Jul 11	14	4	11	6
Jul 25	13	5	16	2
Aug 8	13	4	14	4
Aug 22	9	9	17	1
Sep 5	13	5	14	3
Sep 17	3	14	15	2
Oct 3	8	9	14	3
Oct 17	11	5	16	0
Oct 31	10	7	12	5
Nov 14	12	6	16	2
Nov 28	14	4	14	4
Dec 13	7	10	15	2
Dec 19	12	4	16	0

**Table 2:** Comparison of the qualitative performance of the PCR and larval culture for detecting *Strongylus vulgaris* in the adult horses in the study

	PCR		
	Positive	Negative	Total
Larval culture			
Positive	294	21	315
Negative	100	46	146
Total	394	67	461

The results of McNemar’s test for paired proportions are—McNemar’s test: *χ*^2^ = 153.433, *P* < 0.0001

### *Strongylus vulgaris* qPCR

The PCR results are presented in Fig. 1 and Table 1. Table 2 presents the results of the McNemar test for comparisons between the coproculture and PCR results. The qPCR detected 84.5% of samples as positive, which was statistically higher than the PCR. There were no statistical associations between mean Ct values and season, time point, age, or parturition status.

### *Strongylus vulgaris* ELISA

ELISA results are presented in Fig. 1. There were no statistical differences in *S. vulgaris* ELISA values between seasons or collection points, or in regard to parturition or age in the adult horses.

Foal ELISA values are presented in Fig. 3. Antibody concentrations steadily decreased until 16 weeks of age, and then became highly variable and increased drastically, both as a percentage of the mare’s ELISA value and as a percentage of the positive control (*P* = 0.0455).

### Worm counts in foals

#### Cyathostomins

Cyathostomin worm and encysted larval counts are presented in Table 3. Mucosal cyathostomin stages identified were LL3/L4s and counts ranged from 0 to 6000 larvae and were significantly lower in the younger foal group (*P* = 0.0303). In the group of older foals, mean larval counts were significantly higher in colts than in fillies (*P* = 0.0366).

**Table 3:** Mean cyathostomin worm burdens in the two age groups of foals in the study

Age group	Cecum	Ventral colon	Dorsal colon	Overall luminal counts
Luminal cyathostomins				
4–6 months	144 (142)	2378 (3706)	1241 (1192)	3733 (4925)
8–10 months	800 (779)	33,400 (31,340)	10,775 (15,458)	44,975 (46,060)
Encysted cyathostomins				
4–6 months	1611 (1799)	1278 (1277)	444 (768)	3333 (3029)
8–10 months	6125 (4990)	3875 (4768)	375 (479)	10,375 (7687)

Standard deviation in parentheses

#### Strongylins

Counts of non-cyathostomin parasites are presented in Table 4. Total migrating *S. edentatus* larval counts were highest in fillies (*P* = 0.0103) but did not vary significantly between the two age groups. Total migrating and luminal *S. vulgaris* counts were highest in colts 6–8 months of age, and ranged from 0 to 67 worms, with the majority occurring in the CMA (0–62). ELISA and FEC values were not statistically significantly associated with either counts of migrating or luminal stages of *S. vulgaris* in these foals.

**Table 4:** Mean non-cyathostomin worm burdens in the two age groups of foals in the study

Age group	Migrating stages	Luminal stages
*Parascaris* spp.		
4–6 months	N/A	8 (28)
8–10 months	N/A	3 (14)
*Anoplocephala perfoliata*		
4–6 months	N/A	1 (1)
8–10 months	N/A	10 (16)
*Strongylus vulgaris*		
4–6 months	11 (9)	0 (0)
8–10 months	27 (26)	6 (12)
*Strongylus edentatus*		
4–6 months	11 (23)	0 (1)
8–10 months	35 (10)	1 (1)

Standard deviation in parentheses

#### Other parasites

*Parascaris* spp. counts were highest in colts 4–6 months of age, but no differences were found between fillies aged 4–6 months and 6–8 months (Table 4). *Anoplocephala perfoliata* counts were highest in foals 6–8 months of age and ranged from 0 to 43 worms.

## Discussion

This study is the first comprehensive study of equine strongylid parasite transmission in North America. Furthermore, we have detailed the dynamics of *S. vulgaris* infection through coproculture, PCR, ELISA, and worm count data. Finally, this is the first study since 1974 to investigate the presence of PPR in horses.

Our results demonstrate a lack of seasonal differences in strongylid egg shedding among the mature horses. As outlined in the introduction, this is in stark contrast to both historical and more recent studies, which collectively demonstrated clear seasonal differences [11, 18–22]. However, as mentioned previously, most of these previous studies were conducted in the UK, and since very few studies have been completed elsewhere, these patterns may differ substantially between regions and climates. Recent work with a cyathostomin computer model has suggested widely different transmission patterns between seasons and climates [42, 43], and it is therefore plausible that strongylid egg shedding patterns may differ between climates in a similar fashion. However, it should be acknowledged that the studied population may not be completely representative of managed horses in central Kentucky or elsewhere. Mean strongylid egg counts for the mature horses undulated around 1000 EPG throughout the study (Fig. 1), and this must be considered as unusually high for this age group. This can probably be largely explained by the lack of anthelmintic treatment since 1979, which undoubtedly has led to parasite dynamics very different from those of managed and routinely dewormed horses. Nonetheless, the aim of this study was to investigate parasite shedding seasonality as it occurs naturally in the absence of anthelmintic treatment, which justifies the choice of study population.

This study did not provide any evidence of a strongylid PPR (Fig. 1), which is in agreement with the observations reported by Duncan almost 50 years ago [11]. This is useful information for constructing parasite control programs in breeding operations and suggests that foaling mares do not need strongylid-directed treatments in addition to what is generally recommended for adult horses.

Shedding of *S. vulgaris* was found throughout the study, as reflected by both the coproculture and the PCR data (Fig. 1). Although statistically significant differences were not found between seasons, the data do resemble those described by Duncan in 1974 [11], with more shedding during spring months than at other times of the year. This can be explained by the duration of the *S. vulgaris* life-cycle, which includes a prepatent period of about 6 months. As suggested by Duncan [11], L3 larvae are ingested during the spring, summer, and early autumn, which means that many larvae will be migrating in the mesenteric arteries during the winter months and subsequently complete the life-cycle in the following spring. The shedding of larger numbers of *S. vulgaris* in the older age group has not been previously reported and may be due to differing immune responses between the two age groups. However, given the uneven distribution of horses between the two groups and the relatively small group size, this must be interpreted with caution. The serum ELISA values (Fig. 1) demonstrate a generally high *S. vulgaris* exposure in this herd, which is not surprising given the lack of anthelmintic treatment. The infection pressure is further evidenced by the *S. vulgaris* worm and larval counts in the necropsied foals (Table 4), which indicated that these infections are acquired early in life despite their diets consisting primarily of milk. Previous studies have demonstrated that it can take several months for antibody concentrations to reduce significantly following an effective anthelmintic treatment [44, 45], so even if *S. vulgaris* infection pressure fluctuates during the year, this would be unlikely to translate to a similar variation in antibody concentration. The foals displayed clear evidence of passive transfer of maternal anti-*S. vulgaris* antibodies and mounted their own antibody response beginning at about 20 weeks of age. A previous study from the same herd found a similar pattern [32], although substantial differences were noted between the two studies. In the study reported herein, the passively transferred maternal antibody concentrations were substantially higher, and the subsequent seroconversion occurred 4–5 weeks later compared to the study reported in 2014 [29]. Furthermore, *Parascaris* and *Strongylus* spp. burdens were considerably smaller in the foals in the present study compared to the previous findings [29]. It appears that infection pressures may have been different between the studies, and that weather patterns could represent one explanation of these observed differences. The foal/mare ELISA ratios (Fig. 3) demonstrated that while the concentration of the transferred maternal antibodies was a function of the serum antibody concentration of the corresponding dams, the subsequent antibody production was more variable between foals and, thus, less related to mare antibody concentrations.

In the foals, it is interesting to note the differences between the sexes, with colts shedding higher number of ascarid, *S. westeri*, and strongylid eggs overall (Fig. 2). This has not been previously demonstrated in this herd, and we are not aware of any such findings in other studies. In ruminants, males have been described to be more susceptible to gastrointestinal nematode infection than females [46], but a similar phenomenon is not widely described in horses. We have previously found fillies to harbor significantly higher numbers of *Parascaris* spp. in foals from this population [37], but that did not translate to a similar difference in ascarid egg counts. It should be noted that the colts excreted more strongylid eggs than the fillies at 2–8 weeks of age. As the shortest egg reappearance period reported for equine cyathostomins is 6 weeks [14], these findings must reflect coprophagy, and they suggest that colts may be more engaged in such activity than fillies. These results should be interpreted with caution given the limited number of foals and the uneven distribution of colts and fillies in the study, and these observations could have been affected by single outliers. It is worth noting that we found *S. westeri* egg shedding starting at 2 weeks and continuing until 14 weeks of age. This is in general agreement with Lyons’ original description of the *S. westeri* life-cycle, where he described egg shedding to generally start at 8 days of age and peak at 11–12 days of age, with a proportion of foals remaining egg count-positive until 20–26 weeks of age [47]. These results also agree with a previous study which demonstrated that *S. westeri* egg counts were positive in foals up until 123 days (17 weeks) of age, after which all investigated foals had fecal egg counts of zero [48].

## Conclusions

This study has provided novel information about parasite infection dynamics in horses kept in a Southeastern US climate. The results demonstrated a lack of seasonal differences of strongylid egg shedding and an absence of PPR in foaling mares but did suggest some seasonal pattern of *S. vulgaris* transmission. Furthermore, the results demonstrated the dynamics of passive transfer of maternal anti-*S. vulgaris* antibodies to foals and the establishment of several parasite categories during the first months of life. These results will be useful for understanding parasite epidemiology in a broader context, and emphasize the value of collecting this type of information from a variety of climatic settings. Certainly, this study demonstrated substantial differences from findings in previous studies primarily in the UK.

## Data Availability

The datasets used and/or analyzed during the current study are available from the corresponding author upon reasonable request.

## Abbreviations

FECs: Fecal egg counts
EPG: Eggs per gram
ELISA: Enzyme-linked immunosorbent assay
PCR: Polymerase chain reaction
IACUC: Institutional Animal Care and Use Committee
CMA: Cranial mesenteric artery
